# Data Resource Profile: The ALSPAC birth cohort as a platform to study the relationship of environment and health and social factors

**DOI:** 10.1093/ije/dyz063

**Published:** 2019-04-21

**Authors:** Andy Boyd, Richard Thomas, Anna L Hansell, John Gulliver, Lucy Mary Hicks, Rebecca Griggs, Joshua Vande Hey, Caroline M Taylor, Tim Morris, Jean Golding, Rita Doerner, Daniela Fecht, John Henderson, Debbie A Lawlor, Nicholas J Timpson, John Macleod

**Affiliations:** 1 Avon Longitudinal Study Parents and Children, Population Health Science, University of Bristol, Bristol, UK; 2 Centre for Environmental Health and Sustainability, University of Leicester, Leicester, UK; 3 Small Area Health Statistics Unit (SAHSU), Imperial College London, London, UK; 4 ALSPAC Original Cohort Advisory Panel (OCAP), University of Bristol, Bristol, UK; 5 Department of Physics and Astronomy, University of Leicester, Leicester, UK; 6 Centre for Academic Child Health; 7 MRC Integrative Epidemiology Unit, Population Health Science, University of Bristol, Bristol, UK

## Data resource basics

This resource profile describes the information about the physical and social environment collected within the Avon Longitudinal Study of Parents and Children (ALSPAC) birth cohort. This includes spatial and temporal information gathered on three generations about:

- area-level built and social characteristics (e.g. density and location of fast-food outlets, crime rates within a neighbourhood);
- exposure measurements (e.g. air pollution concentrations, temperature records);
- participant-reported data directly related to the spaces and places they inhabit (e.g. neighbourhood safety, presence of damp within a home);
- information directly measured from participants (e.g. blood lead and total mercury concentrations, physical activity);
- the location information needed to link these diverse data.

We describe the platform’s previous uses, strengths and weaknesses and access arrangements, emphasizing confidentiality safeguard controls. This profile highlights a particular class of ALSPAC data (with distinct access arrangements) to promote the potential for incorporating physical environment and other spatially-dependent data into research investigations.

### The Avon Longitudinal Study of Parents and Children

ALSPAC is a multi-generational prospective birth cohort study^1^^,^^2^ that has compiled an exceptionally detailed longitudinal resource of directly measured and linked phenotype and ‘omic’ data.^3^ ALSPAC’s eligible sample is defined as all pregnant women living in and around the city of Bristol (south-west UK) and due to deliver between April 1991 and December 1992. Women carrying a total of 20 248 pregnancies were deemed eligible. Of these, ALSPAC has recruited ‘G0’ (generation zero) mothers of 15 247 pregnancies, which resulted in 15 458 ‘G1’ (index generation) fetuses. Of this total sample of 15 458 fetuses, 14 775 were live births and 14 701 were alive at 1 year of age. By April 2018, the G1 index participants had reached young adulthood, with many having children of their own. Recruitment of the third-generation ‘G2’ (children of the G1 index sample) began in 2012 and included recruitment at any age from *in utero* onwards.^4^ By January 2019, over 907 G2 children from over 604 families have been recruited into ALSPAC.

**Figure 1.**
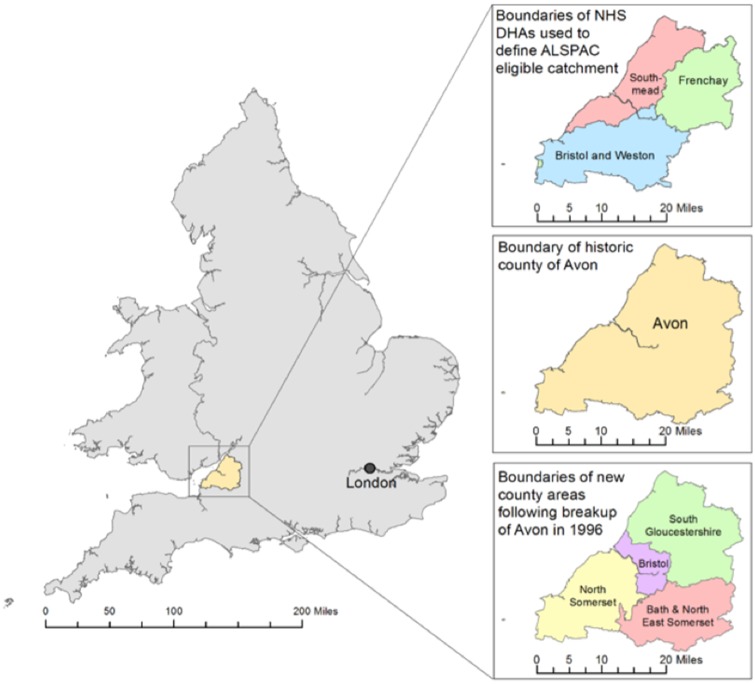
The ALSPAC Eligible Study Area within the UK: illustrating the NHS District Health Authorities (DHAs) used to define: the ALSPAC catchment area; the historical county of Avon; and the four authorities formed following the breakup of Avon. Contains Ordnance Survey, Office of National Statistics and National Records Scotland data © Crown Copyright/database right 2014.

The ALSPAC catchment was centred around the city of Bristol, 106 miles west of London. It comprised three health administration districts within the South-West Regional Health Authority that later became the ‘Bristol & District Health Authority’ (Figure 1). This area largely overlapped with the County of Avon, which was restructured in 1996 into the City of Bristol and the counties of Bath & North East Somerset, North Somerset and South Gloucestershire. Local employment is largely tertiary sector (i.e. commercial and government services), although Bristol is noted for high-tech aerospace manufacturing and agriculture and food/drink production. The area has a temperate climate and its geology is predominantly sedimentary (carboniferous limestone). Local natural resources include coal, iron, lead and zinc, which have been mined locally for up to 2 000 years.^5^Table 1 describes differences between the City of Bristol, the wider metropolitan area and the whole of England and Wales in terms of population growth, density, age, ethnicity and economic activity. More detailed demographic and environmental assessments are compiled in UK government reports^6^^,^^7^ and census-based reports.^8–10^

**Table 1.** Comparison of a selection of population characteristics in the City of Bristol, the wider metropolitan area and the whole of England

	Counties overlapping with the ALSPAC catchment area				England & Wales
	Bath & North East Somerset	Bristol, City of	North Somerset	South Gloucestershire	
Population					
1991	150 682	354 791	169 608	212 558	47 595 169
2001	169040	380615	188564	245641	52041916
2011	176016	428234	202566	262767	56075912
2011 urban (%)	78.92	100	81.62	86.92	81.54
2011 rural (%)	21.08	0	18.38	13.08	18.46
2011 density (no. of people/hectare)	5.1	39.1	5.4	5.3	3.7
Age, ethnicity and economic activity					
2011 mean age	40.3	36.5	42.6	39.8	39.4
2011 White residence (%)	94.6	84.0	97.3	95.0	86.0
2011 aged 16-74 and economically active (%)	68.7	70.6	70.6	74.4	69.7

*Source:* Office for National Statistics (ONS). ONS Crown Copyright Reserved.

## Data collected

Data in the ALSPAC resource can be linked with physical and social environment records using geocoded databases recording participant life-course location.

### A geocoded database for the ALSPAC cohort

Central to ALSPAC acting as a platform for geospatial and temporal research is the database of participant addresses and other key location information (e.g. school addresses). ALSPAC has maintained an administrative database of participant address details since recruitment, and invests considerable resource in maximizing the completeness of this record (see Supplementary materials, available as Supplementary data at *IJE* online).

#### Geocoding G0, G1, G2 participant residential addresses

Participants’ address records have been cleaned and geocoded at the property central coordinate (centroid) and postcode centroid level to 1-m Easting and Northing cartesian coordinates. In the UK, postcodes group properties into small clusters containing an average of 15 properties (range 1–100). We used coordinate data in Ordnance Survey’s AddressBase Plus product to geocode properties, and the Office of National Statistics Postcode Directory (ONSPD) to geocode postcodes.

#### Geocoding G1 participant school addresses

G1 school attendance records from the English National Pupil Database (NPD) attainment and census databases were combined to create a record of school attendance from age 4/5 to 18 (∼85% of G1 index participants are linked to their NPD record). School postcode centroids have been geocoded to 10-m coordinates (see Supplementary materials, available as Supplementary data at *IJE* online). This geocoded database will be iteratively updated over time and could be extended to new participant-related locations (e.g. registered general practice, workplace) or, in theory, dynamic location models based on personal sensor data.

### Participant self-reported and study-collected physical environment and location-based measures

The ALSPAC database contains participant self-reported, teacher-reported and study fieldworker-assessed variable data and biological measures related to the home, school and natural and physical environment (including participant neighbourhood). These data have been collected at individual and household levels and are summarized in Table 2. G0 mother-reported data about the home they and their G1 child live in (e.g. degree of damp and mould, new furnishings and carpets) have been collected at six time points: antenatally, and at G1 age 8 months, 21 months, 33 months, 61 months and 85 months. There is also self-reported information on modes of transport and occupational history of the G0 mother and her G0 partner. ALSPAC and other fieldworkers have collected air pollution data from subgroups of the G0/G1 homes using sensors (e.g. Palmes NO_2_ sensor) and through collection of biological samples (e.g. venous blood carboxyhaemoglobin and methaemoglobin readings). Biological measures of lead, cadmium and total mercury and other elements were measured in G0 maternal blood early in pregnancy, in G1 umbilical cord tissue samples (samples collected by midwives) and in G1 children’s blood (for lead only) at age 30 months (collected in the Children in Focus assessment clinic). ALSPAC is testing ‘internet of things’ approaches^11^ and the use of personal air pollution sensors and location tracking sensors (unpublished), and is collecting information using head cameras to study interaction between G1 parents and their G2 offspring at home, in the garden and at other locations.^4^

**Table 2.** Summary of ALSPAC participant reported and study collected measures

Assessment name	Method	Type	Description	Sample n (units)	Time point
BRE Study^a^	Fieldwork by BRE	Sensors	Formaldehyde, toluene and other volatile organics Nitrogen dioxide Fungi, house dust mite and bacteria Temperature and humidity	174 (homes)	Antenatal to G1 6 m
Heavy Metals	Antenatal clinic ICP-DRC-MS^b^	Biosample	Lead, cadmium and total mercury concentrations (venous blood)^c^	4285, 4286, 4134, respectively (G0)	Antenatal
	Maternity hospital ICP-OES^d^/ICP-MS^e^		Lead, cadmium and total mercury concentrations (cord tissue)^f^	889, 2832, 2600, respectively (G0/G1)	Birth
CiF NO2 Study	Tubes sent by post	Sensor	NO_2_ measured using inside child’s bedroom (Palmes tube)	1200 (G1)	G1 3-12 m
			NO_2_ outside the front of the house (Palmes tube)	700 (homes)	
CO Study	Fieldwork	Sensor	CO indoor background (Draeger diffusion tube) CO exhaled breath (Bedfort EC50 ToxCO breath CO monitors)	80 (homes)	G1 96-124 m
		Biosample	Carboxyhaemoglobin and methaemoglobin levels (venous blood)		
CiF Alveolar CO Study	Focus clinic	Sensor	Alveolar carbon monoxide concentrations (Bedfort EC50 ToxCO breath CO monitors)	1219 (G0)	G1 12 m
Heavy Metals	Focus clinic AAS^g^	Biosample	Blood lead concentration in G1 children (venous blood)	582 (G1)	G1 30 m
Indoor Environment	Self-report by mothers	Questionnaires	Variables including: Type of housing, including storey Degree of damp and mould in each room Frequency with which windows were opened in summer/winter Type of heating and cooking used Wallpapering, painting, new furniture or carpets and in which rooms Household/occupational/hobby chemical use Noise	Various *n* depending on time point	Various times from pregnancy through childhood
Outdoor Environment & Lifestyle	Self-report by mothers	Questionnaire	Variables including: Traffic density on the road Modes of transport Time spent outdoors Mothers and fathers/partners occupational history (coded to SOC90) neighbourhood quality	Various *n* depending on time point	Various times from pregnancy through childhood
School Environment	Head teacher report	School-based Questionnaire	Variables including: Distance to road Noise Building and facility quality	Head teacher 1017 and 1004 responses; class teacher 1339 and 1435 class teachers	School years 3 and 6

m, months.

a The Building Research Establishment (BRE) study was of 174 homes (quasi-random selected) and each assessed over a 12-month period.

b ICP-DRC-MS, inductively coupled plasma dynamic reaction cell mass spectrometry.

c In addition: selenium (Se).

d ICP-OES, inductively coupled plasma optical emission spectrometry.

e Elements (except Pb) were assayed by ICP-OES (*n* = 2005), except for Se and Hg, which were measured by atomic fluorescence techniques (hydride generation and cold vapour, respectively); the final 911 samples were assayed for these elements plus Pb by ICP-MS.

f In addition: Se, Mg, Ca, Cr, Mn, Fe, Co, Ni, Cu, Zn, Sr, Mo, Sb, K.

g AAS, atomic absorption spectroscopy.

### Linkage to third party physical environment records

The ALSPAC geocoding can be used to link any information recorded against a geolocated point or area into the ALSPAC databank. Whereas the possible linkages are numerous, these fall under a range of broad data domains (e.g. geological, housing stock characteristics) that can inform assessment of a range of exposure types (e.g. ambient residential exposures such as potential domestic radon gas exposure from underlying geology) (see Table 3).

**Table 3.** Domains of geolocated information and types of exposure that are, or could potentially be, linked to and evaluated with ALSPAC data

Domains	Types of exposure
Meteorological, climate and associated emissions (e.g. ultraviolet radiation) Outdoor ambient air quality (e.g. industrial air pollutants, pollen count) Water quality (e.g. drinking water additives and quality) Green space, blue space and land use (including plant species) Geological (e.g. radiation) and topographical (e.g. altitude, aspect and hydrology) Noise, vibration, radiation and electromagnetic fields	Ambient residential exposure (e.g. air pollution, noise levels) Ambient occupational exposure (e.g. noise levels) Indoor residential exposure (e.g. indoor air pollution) Modelled commute exposure (e.g. air pollution, pollen count) Other residential exposures (e.g. water quality, radiation) Accessibility to services and amenities (e.g. green space) Extent and density of built environment Active transport connectivity

These data can be collected *in situ* (local point measurement, such as air pollution sensor data). Alternatively, the data can be modeled using: survey data (e.g. maps of radon potential); satellite remote sensing data (e.g. vegetation measured from land cover type); spatio-temporal data (e.g. time-resolved air pollution exposure maps); or environmental exposure proxy data (e.g. distance to nearest main road; traffic intensity (count) multipled by distance to the nearest main road). Consequently, records can be generated locally, nationally or internationally and by a diverse range of organizations. Table 4 provides illustrative examples of these data sources and includes a more detailed exemplar summarizing the potential linked data inputs that could be used to model NO_2_ exposure in the ALSPAC catchment area from road and local sources using a methodology established previously to model particulate matter exposure.^12^

**Table 4.** Illustrative examples of physical environment data that could be linked to ALSPAC, including a summary of the potential sources to inform NO_2_ modelling

**Table 4a** Sources of physical environmental data	**Table 4b** Illustrative ambient outdoor air pollution data with potential to inform NO_2_ exposure modelling
National ‘static’ maps and inventories DEFRA annual average background air pollution maps national atmospheric emissions inventory (NAEI) Time-varying, spatially-gridded validated governmental / agency data Met Office meteorological data ECMWF CAMS modelled atmospheric data Nationally distributed time-resolved point measurement data DEFRA AURN measured air quality data CEH COSMOS-UK soil moisture measurement network data Local government repositories Bristol environmental survey data^a^ County road traffic count data^b^ Research data (one-off measurement, modelling campaign data, and sustained monitoring in selected locations) NERC-funded projects^c^ Crossover data repositories UKEOF funded by NERC and DEFRA) Open satellite data downloads NASA MODIS aerosol optical depth Model data estimating the natural and physical environment ADMS-Urban air pollution model (commercial software) CMAQ (open source software) Statistical models estimating exposures from multiple sources Land use regression models 3D mapping of the built and natural and physical environment Google Earth 3D Building Data Bluesky National Tree Map	Model data: A city-wide (approx. 30 km) scale 3-hourly data from satellite-driven model ECMWF CAMS (NO_X_) DEFRA hourly air pollution *in situ* point measurements (NO_X_) (from 1990 for some pollutants) National Atmospheric Emissions Inventory on annual average major pollution sources and roads emissions estimates (from 2001) County council road traffic data Validation data: City council historical measured diffusion tube data on NO_2_ exposure over two 4-week periods and ALSPAC data on 700 homes^16^
	Chemicals ingested with food or otherwise, or skin exposure to chemicals, are excluded as they are unlikely to be available through straightforward linkage to external records (although there is potential to map probabilities of some of these exposures). Assessments of indoor air pollution exposure must be measured and/or modelled individually (future developments may make indoor exposure modelling possible by combining ambient outdoor air pollution levels with other determining factors such as smoking habits, cooking practices, ventilation, year of house build etc)

a Bristol City Council data can be accessed here: [https://opendata.bristol.gov.uk/explore/].

b Road traffic count data can be accessed here: [https://www.dft.gov.uk/traffic-counts/index.php].

c NERC-funded research data can be accessed here: [https://csw-nerc.ceda.ac.uk/].

### Linkage to social and built environmental records and administrative geographical areas

Participant postcodes (across the UK) have been mapped onto official UK areal units (geographies) using the National Statistics Postcode Lookup (NSPL) which provides administrative, electoral, census, health and Eurostat geographies at [https://www.ons.gov.uk/methodology/geography/ukgeographies], as well as neighbourhood and material deprivation index scores and their sub-domains [Townsend Index Scores and Indices of Multiple Deprivation (IMD)]. These have been mapped (where available) to all data collection points, across G0, G1 and G2 (Supplementary materials, available as Supplementary data at *IJE* online). Pseudonymized versions of these geographies can be used for multilevel modelling, and the geographies themselves can be used to link national and locally collected data such as the Bristol Quality of Life Survey [https://www.bristol.gov.uk/statistics-census-information/the-quality-of-life-in-bristol].

For researchers looking to investigate neighbourhood characteristics or questions pertaining to ‘ecological’ or ‘built’ environments, ALSPAC can calculate Euclidean distances and transform them into usable and privacy-preserving categorical exposures or confounders (e.g. distance to nearest GP surgery).

### Linkage to the existing ALSPAC databank and new data collection

ALSPAC has collated an extensive body of multigeneration life course and genetic data, sampled at frequent intervals and augmented with linkage to routine primary and secondary health care records and social administrative records. The databank can be browsed via the study website where comprehensive records can be searched through: data dictionaries [http://bris.ac.uk/alspac/researchers/data-access/data-dictionary/]; a variable search tool [http://variables.alspac.bris.ac.uk]; and via third party search platforms such as CLOSER Discovery [http://discovery.closer.ac.uk/].

ALSPAC welcomes proposals to collect new data, including environmental data. New data collection strategies can potentially use innovative methods and quantitative and qualitative approaches (e.g. questionnaire, study assessment clinics, biological samples, face-to-face interviews), and could include linkage to routine records or linkage via sensors. Please contact the ALSPAC Executive if considering new data collection activities, via e-mail [alspac-exec@bristol.ac.uk].

### Data processing pipelines and data quality

**Figure 2.**
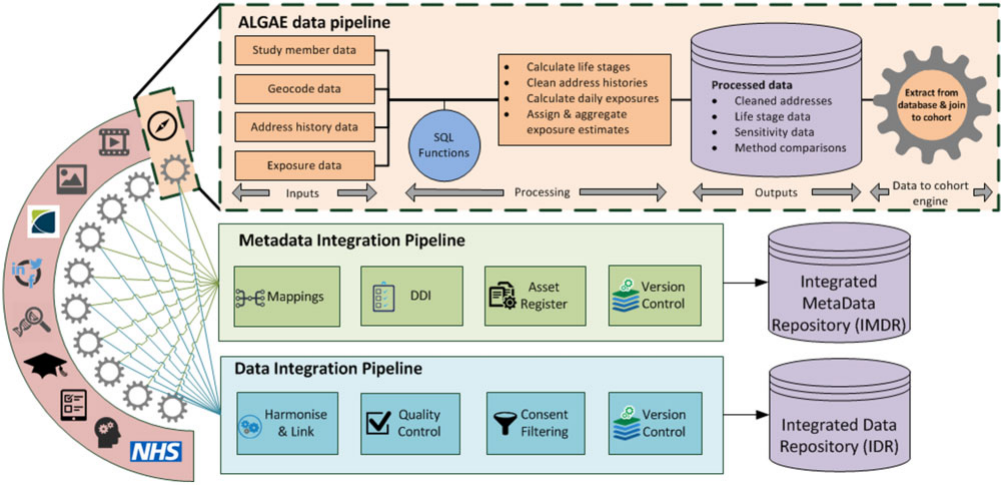
PEARL’s generalized data model illustrating the extraction of radon exposure data, their subsequent transformation and,assignment to cohort participants using the ALGAE ‘data-to-cohort engine’.

The Project to Enhance ALSPAC through Record Linkage (PEARL) [http:// bristol.ac.uk/population-health-sciences/research/groups/pearl/] has established a data model for integrating, cleaning, processing and documenting data into combined ‘research ready’ data outputs (Figure 2). For any given data input type, the model has: (i) a distinct pipeline that captures data using ‘extract, transform, load’ processes that attempt to assess and quantify error, while maximizing potential for future use through capturing as many data as possible on as wide a coverage of the ALSPAC sample as possible; (ii) a ‘data-to-cohort’ integration engine that makes use of standardized tools/measures to link extracted data to participants; and (iii) integration pipelines, creating ‘research ready’ data that fulfill governance expectations and have accompanying provenance and documentary metadata.

For the integration of location-based data, ALSPAC has adopted the ‘ALGorithm for Generating Address Exposures’ (ALGAE) protocol as our integration ‘engine’ (i.e. the process by which raw exposure data are transformed and processed into data which are compatible with the wider ALSPAC resource). This protocol—a generic solution suited for all longitudinal population studies (LPS), developed by the Small Area Health Statistics Unit and ALSPAC—allows ALSPAC to link geolocated data to participants and calculate individual-level exposures at key life stages [https://smallareahealthstatisticsunit.github.io/algae/]. ALGAE can, at an individual level: (i) determine the start/end dates of participant life stages (e.g. pregnancy trimesters); (ii) systematically clean and reconstruct address histories; (iii) calculate daily exposures and assign exposure estimates; and (iv) aggregate exposure estimates over life stages. Thus ALSPAC has a consistent approach for generating cleaned address histories and life stage boundaries, and can provide data quality metrics to research users (such as sensitivity data quantifying data cleaning and method comparisons e.g. cleaned vs not cleaned addresses).

### Maintaining participant confidentiality and acceptability

It is vital that ALSPAC’s data sharing is acceptable and transparent to participants and is compliant with relevant legislation. We have consulted participant representatives (ALSPAC Original Cohort Advisory Group, OCAP) to understand participant views on the use of spatial data in ALSPAC research (Panel 1), and OCAP members are co-authors on this publication.

**Panel 1. ALSPAC’s use of participants’ location data: a participant perspective**

**Introduction:** ALSPAC data managers consulted the Original Cohort Advisory Panel (OCAP) aiming to understand: participant views on personal location data, whether this research is viewed as important and within the scope of the study; and if participants had concerns or perceived there to be risks to this type of research. Established in 2006, OCAP currently comprises around 30 participants (aged 25–27).

**Methods:** In late 2017, data managers attended an OCAP meeting (members unable to attend were able to provide written comments). To encourage discussion, the data managers presented hypothetical research scenarios that described sharing approximate location (e.g. 1 km^2^ area), specific locations (e.g. home or school addresses) and exact location (e.g. GPS tracking). Two participants summarized OCAP views for this publication, with this text approved by the full group.

**Results:** Regardless of the scenario presented, there was consensus that this type of research is important, particularly where the potential to improve public health was clear. Research using personal location data was perceived as different from other research, but within scope of the study. Several participants mentioned that the data that have already been collected should be made the most of. Many of the concerns raised could be addressed by standard safeguards that are in place for other types of ALSPAC data: for example, issuing contracts for data sharing, enforcing sanctions for misuse, and encryption of data. There was some discussion around the feedback of results to participants. Again, clarifying standard ALSPAC procedures resolved the questions; participants would not expect personal return of results and the benefits would be felt by wider society. A small number of participants expressed concerns about aspects of sharing approximate and specific location data. In general, the group were comfortable with the sharing of approximate location data and this was not perceived as being as personal as the other location data under discussion. However, a few participants remained concerned about the potential for identification where cell sizes were small. With regard to sharing specific location data, there was some indication that certain locations are perceived as more sensitive than others. For example, some participants expressed that they were more comfortable for their school address to be shared than their home address, owing to the number of other students at the school (though the question of small cell sizes arose again). There was some concern that the sharing of multiple locations would raise the risk of identification, and that conceptualizing certain locations as ‘historical’ is inappropriate as they may still be current for participants and their families. The biggest concern in relation to sharing specific location data was that multiple datasets could be linked through common variables, thus making identification more likely. Of course, this problem is not unique to ALSPAC, but also applies to many other longitudinal studies. Some participants felt reassured knowing that only bona fide researchers would be given access to these data. However, this issue remained a significant concern for a small number of participants.

Across the group there was less consensus with regards to collecting and sharing exact location-tracking (e.g. Global Positioning System) data. Some participants immediately found this acceptable whereas others did not. It was recognized that, as this would involve new data collection, participants could choose not to take part in this. One participant highlighted that new data collection would be scrutinized by an ethics committee and that their concerns lay more in the secondary access to these data. Some perceived harms were expressed by the group (such as the use of these data in legal cases). However, there was a general sense that many participants already face these risks in their day-to-day lives owing to commercial collection of location data. Indeed, it was suggested that participants might find this type of data collection more acceptable because of familiarity with this type of data collection. In general participants were not concerned by sharing events (e.g. that they passed a certain natural feature) but some had reservations about sharing the location (e.g. that they were on a particular road when they passed it). Some participants had particular concerns when it came to these data being connected to their children. Despite seeing the value in sharing these exact location data and perceiving it as within scope, there remained some concerns, and it was not always easy for participants to rationalize or articulate why the idea did not sit comfortably with them.

**Conclusions:** Five key issues came to light during the overall discussions: (i) the suggestion of using a split processing approach (as described in the main article text) was generally well received and preferred across a majority of scenarios; (ii) separately, there seemed to be a general preference for steps in research that involve processing the personal location data to be done in-house at ALSPAC, though there was also recognition of the significant burden this would place; (iii) in general, participants wanted to know that this type of research is taking place; (iv) in a majority of research scenarios, some type of consent process was expected, with an opt-out campaign receiving generally positive views and being thought of as in keeping with previous campaigns in ALSPAC (e.g. for recall by genotype studies); v) the extent to which personal location data such as addresses are conceptualized as data rather than as a means for participation needs to be carefully addressed. Overall, there was consensus that the types of research enabled by use of personal location data would be important and within scope for the Study. A majority of participants seemed to agree that use of personal location data was acceptable given the safeguards that could be put in place, and that the benefits outweigh the risks. Specific concerns differed between scenarios, suggesting that the safeguards that are put in place could vary in complexity on a case-by-case basis. The sharing of approximate and specific personal location data was arguably more acceptable than sharing exact location-tracking data. However, the discussion reveals that participants are at least willing to consider this option also. Underpinning the discussions was a sense of trust placed in ALSPAC by its participants.

Participants’ views have been integral to shaping the data access policy for sharing location data (Panel 2) and identifying appropriate safeguards. The resulting access policy includes controls developed around the ALSPAC ‘Data Safe Haven’ framework^13^ which incorporates: social controls (e.g. data access contracts); information security safeguards; and technical/data management controls (e.g. disclosure checks). The approach taken is for ALSPAC data managers to efficiently facilitate proposals with greater disclosure risk in a manner that enables the science while protecting participant confidentiality.

**Panel 2. Extract from ALSPAC Access Policy relating to the safeguarding of geospatial data**

Complete postcode data are not usually made available; rather, the very broad first digits of postcodes are released, or information derived from these (e.g. household quintile of Indices of Multiple Deprivation at the time of data completion). However, we recognize that there are times when this information is important for deriving variables, such as for spatial research projects. In these circumstances, we will work with the researcher to produce their derived variables, either conducting the work in-house or using a modified version of the ‘Split-Stage’ Protocol, as follows:

Stage 1: The researcher will be provided with a limited dataset containing postcode and any other essential data. To protect the identities of participants, the genuine participant postcodes will be masked by including other, randomly selected, genuine postcodes and synthetically created essential data.

Stage 2: The researcher will use this dataset to write syntax to generate true derived variables.

Stage 3: The researcher will send encrypted copies of the derived variables to the Study Team, and, upon receipt, delete all copies of the original Stage 1 data.

Stage 4: The ALSPAC Data Team attach the derived variables to the remaining requested ALSPAC information, change the case ID and return this file to the researchers. The derived variables will be checked for disclosure risk and may be processed to a less granular level (the means to achieve this will be discussed and agreed in advance).

IMPORTANT points to consider for projects requesting spatial data:

- Requests for specific geographies may be denied in cases where it is believed participants’ disclosure may be at risk.
- Exact address or complete postcode data will not be provided under any circumstances. Instead a range of derived administrative boundary variables are available as outlined in the data dictionary.
- Each proposal will be judged uniquely on its own merits and disclosure risk profile.
- Previous provision of geographical data are not a guarantee of future provision.
- As a condition of submitting a proposal that includes ALSPAC spatial data, a researcher will be required to include detailed information on the reasoning and methodology behind the requested geography to justify the choice, and to specify why the selected spatial resolution is appropriate for the research question. For instance, in the case of high-resolution geographies being requested, the Executive require justification as to why smaller resolution data are not acceptable.
- Data provided with the highest-resolution geographies (often, pseudonymized Lower Super Output Area) may contain many cases reverted to missing due to low unit population counts. Therefore selecting variables with the highest resolution possible can be counter-productive to research.
- The *ad hoc* method of address data management has permitted a database with extremely high temporal accuracy. However due to historical database errors, and individual level differences in reporting address movement, there will inevitably be a small number of cases that have no address data at certain time points. These missing cases should not greatly affect research that uses additional ALSPAC data, as there is understandably a very high correlation between address accuracy and questionnaire/clinic responses.

Ethical approval for the ALSPAC study was obtained from the ALSPAC Ethics and Law Committee and Health Research Authority research ethics committees.

## Data resource use

A subset of the >2000 ALSPAC academic papers have been reliant on geocoded data or the use of geospatial techniques, and many others have used location-based information as covariates (e.g. adjusting for social position using IMD) or have used geographical areas to support multilevel modelling (e.g. Morris *et al*. 2016).^14^ Examples include (for additional examples see Supplementary materials, available as Supplementary data at *IJE* online):

- investigations: considering relationships between domestic exposures and maternal and child health symptoms/outcomes, identifying associations between: household chemical product use and child wheeze;^15^ and NO_2_ from household sources and infant’s health symptoms.^16^ Validation investigations estimating electromagnetic radiation exposure to pregnant mothers showed that exposures from specific equipment were dominated by the configuration of the home electrical wiring (which cannot be calculated without actual measurement within the home);^17^
- investigations of associations of prenatal lead, mercury and cadmium exposure with indicators of child development (maternal blood,^18^ cord tissue^19^), and of child blood lead with school performance.^20^ An alternative ‘exposome’ approach has been used to identify associations of a suite of exposures to a key child development skill;^21^
- assessing the impact of particulate matter air pollution exposure on gene expression: finding that PM_10_ exposure in early life affects methylation of the CpG cg21785536 located on the EGF Domain Specific O-Linked N-Acetylglucosamine Transferase gene;^22^
- identification by ALSPAC of genetic variation in blood lead and selenium content.^23^^,^^24^ Genomic investigations have identified how genetic traits have the potential to influence the domestic/personal environmental exposures, for example: where genetic propensity to armpit odour was linked to deodorant use^25^; and an association between a single nucleotide polymorphism (SNP) in the oxytocin receptor gene to features of the maternal diet;^26^
- investigations assessing associations between health outcomes and workplace exposures. These indicated an association between paternal occupation and subfertility^27^ and showed some weak evidence that certain maternal occupations were associated with low birth weights^28^;
- investigations considering the impact of residential location and residential movement/migration on health and social outcomes, identifying associations between: residential rurality and diet;^29^ the impact of underlying confounding factors to explain previously identified associations between residential movement and cannabis use,^14^ residential stability and poor mental health;^30^ and the impact of major life events on residential mobility;^31^
- investigations considering movements between places (e.g. the journey from home to school); identifying associations with fast-food consumption^32^ and the role of mode of travel choices on activity levels;^33^
- conducting methodological work to develop environmental exposure modelling techniques within longitudinal research studies, including modelling particulate matter (PM_2.5_, PM_10_) exposures and CO2 exposures;^12^^,^^34^
- neighbourhood measures (e.g. IMD) used to inform purposeful sampling strategies in nested methodological randomized control trials^35^ and qualitative studies;^36^
- ALSPAC phenotype data that have been spatially mapped to inform local health service planning;^37^ and
- ALSPAC informing methodological research: (i) considering whether the manner in which neighbourhood boundaries are drawn aids the subsequent interpretation of findings;^38^^,^^39^ (ii) making contributions towards understanding the quality of sampling methods,^40^ survey methods and evidence^41^ and deriving location-based information from study datasets;^42^ and (iii) testing the feasibility of collecting exposure data within an LPS.^43^

## Strengths and weaknesses

The primary strength of this resource is ALSPAC’s ability to link spatially indexed data to the ALSPAC databank. Our geocoding extends across the life course, from pregnancy (allowing assessment of *in utero* exposure) to date. Geocoded residence history has been supplemented by school location and could be extended to other locations. ALSPAC supports location-based research through pre-emptively building files of commonly requested information and through bespoke linkages to new location-based data. The security controls needed to protect participant confidentiality could be considered a weakness (given they place restrictions on data sharing); yet, our ‘Data Safe Haven’ approach typically allows research to occur without a substantial loss of data specificity, while retaining participant acceptability.

ALSPAC’s regional design is advantageous: as participant clustering provides opportunities to assess locality-based effects (e.g. local geographical mobility) and is specific enough to enable methodological approaches such as multilevel modelling using small-scale geographies and conceptual studies assessing area effects. Conversely, ALSPAC in isolation would not be well suited to studying issues relating to national variation.

Geocoding quality depends on the quality and completeness of participant location information, which is poorer where participants are lost to active follow-up. Given that loss to follow-up is socially patterned, it is likely that participants with the most dynamic movement history (e.g. those in unstable accommodation or migrating to find employment) have disproportionately poorer quality location data. Despite these weaknesses, quality is inherently strong among those directly providing data (where it is likely we have their correct address) and the weaknesses above are to some extent mitigated through our tracking and tracing strategy (i.e. independent collection of location records), the collection of address information through record linkage and the potential to use statistical mechanisms to address missing (not at random) information.

## Data resource access

The ALSPAC databank is accessible as a managed-access resource for the international bona fide research community. Prospective data users are encouraged to: (i) browse the catalogue of existing projects [http:// bristol.ac.uk/alspac/researchers/publications/]: data use is non-exclusive and it is the applicant’s duty to maintain awareness of duplicate or overlapping initiatives; (ii) consider the ALSPAC data access policy^44^; and (iii) apply for access [https://proposals.epi.bristol.ac.uk/]. Standard geolocated data (e.g. IMD, urban/rural status, pseudonymized geographies for multilevel modelling) are available at each data time point. Selected subsets of location-based data are available via the UK Data Archive.^45^ Those considering bespoke linkages of spatially indexed information should contact PEARL who manage ALSPAC data linkages [alspac-linkage@bristol.ac.uk]. All applications are assessed for compliance with ALSPAC’s governance and third party data use arrangements. Data users are required to return newly generated or derived data along with rigorous metadata for future reuse in ALSPAC. All users must abide by information security and governance requirements and uphold participant confidentiality [http://www.bristol.ac.uk/alspac/researchers/access/]. Published outputs are reviewed for conformance to a publication checklist [http://www.bristol.ac.uk/media-library/sites/alspac/documents/alspac-publications-checklist.pdf]. ALSPAC withholds the right to request changes to publication to address risks relating to participant disclosure or bringing the study into disrepute.

**ALSPAC as a resource for environmental epidemiology: in a nutshell**

- The ALSPAC birth cohort study has compiled a detailed resource of physical and social environment measures needed to assess relationships between environmental exposures and health and social outcomes.
- ALSPAC is a three-generation cohort. Those eligible include: all pregnant women living in and around Bristol, UK, and due to deliver April 1991–December 1992; their partners (including the biological father); the index children (*n* = 15 247 enrolled); the index child’s offspring and, where they have an offspring, the index child’s partners.
- Environmental data have been collected using: self-report measures by participants and those associated with them (e.g. teachers); participant household assessments (using sensors); assayed biological samples; and linked routine records, based on geocoded participant address records.
- Data include: indoor and outdoor air pollution measurements; noise measurements; neighbourhood quality measures; linked electoral and census areas; and sociodemographic indicators. Further data can be linked from routine records and modelled exposures (e.g. meteorological data).
- Individual-level data are available upon request to the ALSPAC Executive [www.bristol.ac.uk/alspac]

## Supplementary Material

dyz063_Supplementary_Data
