# Supplementary material for: Data Resource Profile: The ALSPAC birth cohort as a platform to study the relationship of environment and health and social factors
Source: Int J Epidemiol. 2019 Apr 21;48(4):1038–1039k. doi: 10.1093/ije/dyz063 (PMC6693884; doi:10.1093/ije/dyz063)
Supplement: dyz063_Supplementary_Data [file dyz063_supplementary_data.pdf]

Data Resource Profile: the ALSPAC birth cohort as a platform to study the relationship of environment and health and social factors.

Andy Boyd[1*], Richard Thomas[1], Anna L. Hansell[2,3], John Gulliver[2,3], Lucy Mary Hicks[4], Rebecca Griggs[4], Joshua Vande Hey[5], Caroline M. Taylor[6], Tim Morris[7], Jean Golding[6], Rita Doerner[1], Daniela Fecht[2], John Henderson[1], Nicholas John Timpson[1,7], John Macleod[1]

Supplementary Materials

1. Additional detail describing participant reported and study collected measures relating to air pollutants

Specific air pollutant measurements in ALSPAC include the following direct measure sub-sample studies:

(i) The Building Research Establishment (BRE) study, funded by the Department of the Environment, used 174 homes each of which was monitored over a 12-month period. These were selected more or less at random at enrolment into ALSPAC at about 3 months of pregnancy. This was a rolling system with about 10 enrolled each month. The measures continued through the 6 months of pregnancy and the first 6 months of the child's life. Measurements included formaldehyde, other volatile organics, nitrogen dioxide, fungi, house dust mite and bacteria as well as temperature and humidity. Each of the homes completed weekly lists of symptoms of all members of the household. From the results, it was possible to identify the major sources of the total volatile organic chemicals (TVOCs) in the home, and thence assess possible health effects on the whole cohort.

References:

- Brown, V. M., Crump, D. R., Gardiner, D., & Yu, C. W. F. (1993). Long term diffusive sampling of volatile organic compounds in indoor air. *Environmental Technology*, *14*(8), 771-777.
- 13686 Brown, V. M., Crump, D. R., Gardiner, D., & Gavin, M. (1994). Assessment of a passive sampler for the determination of aldehydes and ketones in indoor air. *Environmental technology*, *15*(7), 679-685.
- Brown, V. M., & Crump, D. R. (1998). Diffusive sampling of volatile organic compounds in ambient air. *Environmental monitoring and assessment*, *52*(1-2), 43-55.
- Crump, D. R. (1995). Volatile organic compounds in indoor air. *Issues in Environmental Science and Technology*, *4*, 109-124
- Llewellyn, J. W., & Smith, L. M. (1995). Indoor air quality in the UK: the Avon longitudinal study of pregnancy and childhood (ALSPAC). *IEH Assessment on Indoor Air Quality in the Home: Nitrogen Dioxide, Formaldehyde, Volatile Organic Compounds, House Dust Mites, Fungi and Bacteria, Appendix*, *1*, 314-23.

- Farrow A, Taylor H, Northstone K, Golding J. Symptoms of mothers and infants related to total volatile organic compounds in household products. Archives of Environmental Health: An International Journal. 2003 Oct 1;58(10):633-41.

(ii) The Children in Focus NO_2 study was designed to measure levels of exposure to nitrogen dioxide inside the room where the child slept and outside the home. It used households of Children in Focus together with a weighted sample of preterm infants. Mothers were sent 2 Palmes monitoring tubes when their children were aged between 3 and 12 months – one to be mounted inside the room where the study infant slept (n = 1200), and for 700 of these homes, a further tube to be mounted outside the home. The tubes were capped and returned to the study after about 14 days (Finch et al 1995; Farrow et al 1995). The analyses showed that indoor levels were associated with use of a gas cooker and/or hob, cigarette smoking, use of paraffin or bottled gas for heating, the level of nearby traffic and the outdoor measure of NO_2 (Farrow et al 1997).

References:

- Finch, P., Cheyney, B., Allen, J., Farrow, A., & Greenwood, R. (1995). A survey of residential nitrogen dioxide levels in Bristol, UK. In *International gas research conference* (pp. 366-374).
- Farrow A, Preece S. Nitrogen dioxide in infants' bedrooms: a feasibility study for household-based measurements. Environmental technology. 1995 Mar 1;16(3):295-300.
- Farrow A, Greenwood R, Preece S, Golding J. Nitrogen dioxide, the oxides of nitrogen, and infants' health symptoms. Archives of Environmental Health: An International Journal. 1997 May 1;52(3):189-94.

(iii) Carbon monoxide study: This used household monitors which were placed in a room containing at least one gas appliance. Temperature, humidity and carbon monoxide were monitored. Outdoor background levels of carbon monoxide were assessed using diffusion tubes manufactured by Draeger. Eighty homes were monitored over a five-day period. Carbon monoxide levels from exhaled breath were assessed both at the beginning and the end of the study period (using Bedfont EC50 ToxCO Breath Carbon Monoxide Monitors) on all family members. Carboxyhaemoglobin and methaemoglobin levels were measured in venous blood taken from all willing members of the households at the end of the five-day study period. The aim was to determine whether there was any association with asthmatic symptoms. These results have been written up but not yet published.

(iv) The Children in Focus CO study measured mother's alveolar carbon monoxide concentrations using a Bedfont smokelyzer EC50 lung breath monitor during the 12-month Children in Focus clinic. The instrument was recommended by Dr Robert Waller at the Department of Health, and was lent to the project by the manufacturer. An initial pilot study of 38 mothers showed a strong correlation between urinary cotinine and the CO level measured in the afternoon. The main reason for undertaking this measure was as a validation of maternal self-reported smoking

history, her passive smoking reports and exposure to other noxious fumes such as from traffic and gas cookers. The mother was asked to blow into a tube which was linked to the monitor, and the tester wrote down the number displayed on the screen. There are 1 219 valid readings.

Self-reported questionnaire data relating to sources of air pollution

Questionnaire measures related to possible sources of air pollution include details of the following in regard to the home: Degree of damp and mould in each room; frequency with which windows were opened in summer and in winter; whether the windows were double-glazed; ventilation used in the kitchen; type of heating used; type of cooking; whether there had been wall-papering, or painting and in which rooms; whether there had been new furniture or carpets and in which rooms.

Other data concerning the indoor air environment include the frequency with which various domestic chemicals were used in the home, from which a score has been developed and shown to be normally distributed and significantly related to symptoms of wheezing and asthma, as well as reduced birthweight.

References:

- Sherriff A, Farrow A, Golding J, Henderson J. Frequent use of chemical household products is associated with persistent wheezing in pre-school age children. Thorax. 2005 Jan 1;60(1):45-9.
- Henderson J, Sherriff A, Farrow A, Ayres JG. Household chemicals, persistent wheezing and lung function: effect modification by atopy?. European Respiratory Journal. 2008 Mar 1;31(3):547-54.

2. Additional detail describing ALSPAC management of our participant address database

Maintaining a record of participant address

Maintaining a record of participant contact details (including address) is crucial to the success of any LPS. Therefore, substantial resource is invested in ensuring the ALSPAC address database is accurate, and that participants who are 'lost to follow-up' are located and re-engaged with the study. ALSPAC attempt to ensure these location data are updated via regular contact with participants; through linkage to routine records; and via a 'tracking and tracing' program where fieldworkers search publicly available records and attempt to locate participants who are lost to follow-up. Information on our 'tracking and tracing' program has been published previously by Bray et al. 2015.

Accuracy and completeness of the participant addresses

Numerous factors have impacted on the accuracy and completeness of participant address data. These factors include:

1) ALSPAC's historical failure to ask participants for move dates (instead the reported move date is used as a proxy);

2) index participants continuing to provide parental address rather than their true address (e.g. while attending university or travelling overseas);
3) ALSPAC's limited ability to accurately capture interim addresses when we re-contact participants lost to follow-up. Attempts to capture these data are likely to be impacted by recall error, particularly where significant time has elapsed or where participants have had periods of frequent and/or transient addresses.

Further to these, data management challenges have impacted on the quality and completeness of data. For example, during recruitment ALSPAC possessed limited computing resources, and in order to create computer disk space to enrol new participants it was necessary at times to delete 'expendable' information such as historical address records. The impact of this type of record is difficult to quantify.

Procedure for tracing a lost participant (version 1.2 September 2017)

1) 'Lost' participants are notified to a dedicated ALSPAC 'participation' team, who have responsibility for managing relationships with study participants. Participants are notified as being 'Lost' where: i) postal mail is returned by the postal services as 'addressee not known'; ii) where a direct attempt to contact a participant fails (e.g., where a member of the participation team tries to phone a participant); or, iii) where there has been no response for some time (which varies depending on participation history). It is recognised that some of these triggers may not truly reflect that a participant has moved from their recorded address.
2) Alternate contact mechanisms: in the first instance, the participation team attempt to make contact via alternate contact mechanisms (e.g. phone or email). ALSPAC also ask participants to nominate a 'stable contact' – such as a parent – who can be contacted to provide updated contact details in these circumstances. Staff attempt to contact the individual multiple times during both office hours and in the evenings/weekends.

Where this fails:

3) The participants details are searched using online databases which compile the public domain electoral role, phone directories, directories of company directors etc. The database companies used are contracted to the University of Bristol to provide these services: these contracts include clauses designed to protect the confidentiality of participants.

This is supplemented by:

4) Where data usage agreements and ethical agreements permit, ALSPAC have been able to use participant contact details sourced from linked health and administrative records (e.g. the address at which a patient is registered with the NHS). These data are used in line with the protocol agreed with the data owner: typically, the contact details can only be added into the ALSPAC database (and therefore is geocoded and added to the residential address

database) where a participant provides a positive response to a re-engagement attempt.

Where new contact details are found, contact is made to a set protocol. The protocol attempts to re-engage the participant into the study. It also includes information about how the new address was found. ALSPAC staff are trained in the risks associated with handling address data, and particularly for vulnerable participants who have moved for specific reasons.

References:

Bray I, Noble S, Boyd A, Brown L, Hayes P, Malcolm J, Robinson R, Williams R, Burston K, Macleod J, Molloy L. A randomised controlled trial comparing opt-in and opt-out home visits for tracing lost participants in a prospective birth cohort study. BMC medical research methodology. 2015 Dec;15(1):52.

3. Linkage to social and built environmental records and administrative geographical areas

Whilst ALSPAC does not provide raw postcodes (or other disclosive geographies), researchers may request the indicators outlined in Supplementary Materials Table 1. These measures are derived by census output area (2001, 2004, 2007 & 2011) for any research-question relevant time point, such as date of birth, questionnaire completion or research clinic attendance date. Where disclosure risks exist, ALSPAC aggregates scores into quintile or quartiles (e.g. IMD and Townsend index scores) or provides proxies (e.g. for LSOAs, MSOAs and OAs), which can be used for multilevel approaches to analysis.

Supplementary Materials Table 1: Official UK geographies and deprivation indicators linked to ALSPAC at all timepoints.

**Official UK Geographies**	
Administrative Geographies	County
	Country
	Local authority district (LAD)/unitary authority (UA)/ metropolitan district (MD)
	National grid reference – Easting
	National grid reference – Northing
Electoral Geographies	(Electoral) ward/division
	European Electoral Region (EER)
	Grid reference positional quality indicator
	Pan SHA
	Region (formerly GOR)
	Standard (statistical) region (SSR)
	Westminster parliamentary constituency

	Local Learning and Skills Council (LLSC)/ Dept. of Children, Education, Lifelong
Eurostat Geographies	NUTS/LAU areas
	ED positional quality indicator
Health Geographies	Previous Strategic health authority (SHA)/ health board (HB)/ health authority (HA)
	Clinical Commissioning Group (CCG)/ local health board (LHB)/ community health p
	Local Education Authority (LEA)/ Education and Library Board (ELB)
	Primary Care Trust (PCT)/ Care Trust/ Care Trust Plus (CT)/ local health board
	Health Authority ‘old-style’
	‘Old’ Primary Care Trust (PCT)/ Local Health Board (LHB)/ Care Trust (CT)
	Strategic health authority (SHA)/ health board (HB)/ health authority (HA)/ heal
Census Geographies	1991 ward (Census code range)
	1991 ward (OGSS code range)
	1998 ward
	2005 ‘statistical’ ward (England and Wales only)
	2001 Census output area
	Census Area Statistics (CAS) ward
	1991 Census Enumeration District (ED)
	1991 Census Enumeration District (ED)
	2001 Census lower layer super output area (LSOA)
	2001 Census middle layer super output area (MSOA)
	2001 Census urban/rural indicator
	2001 Census output area classification (OAC)
	2011 Census output area (GB)/ small area (NI)
	2011 Census lower layer super output area (LSOA)
	2011 Census middle layer super output area (MSOA)
	Parish (England)/ community (Wales)
	2011 Census workplace zone
	Built-up area
	Built-up area sub-division
	2011 Census rural-urban classification
	National park
Other	LA CODE
	GOR CODE
	PRE 2009 LA CODE
	POST 2009 LA CODE
	Travel-to-work area (TTWA)
Deprivation Indicators*	
	IMD Score
	IMD Income Score

IMD Employment Score
IMD Health Score
IMD Education Score
IMD Housing Score
IMD Access Score
IMD Child Poverty Score
Townsend Index Score

---

*Index of Multiple Deprivation Scores (IMD) derived for Census Area Output Areas as defined in 2001, 2004, 2007 & 2011

4. Data Resource Uses (further examples)

Further examples of work relating to household chemical use include:

- Henderson J, Sherriff A, Farrow A, Ayres JG. Household chemicals, persistent wheezing and lung function: effect modification by atopy?. European Respiratory Journal. 2008 Mar 1;31(3):547-54.
- Farrow A, Taylor H, Northstone K, Golding J. Symptoms of mothers and infants related to total volatile organic compounds in household products. Archives of Environmental Health: An International Journal. 2003 Oct 1;58(10):633-41.

Further examples of work relating to electro-magnetic radiation exposure include:

- Preece AW, Kaune W, Grainger P, Preece S, Golding J. Magnetic fields from domestic appliances in the UK. Physics in Medicine & Biology. 1997 Jan;42(1):67.
- Preece AW, Grainger P, Golding J, Kaune W. Domestic magnetic field exposures in Avon. Physics in Medicine & Biology. 1996 Jan;41(1):71.

Further examples of work relating to metal exposure and child development outcomes include:

- Taylor CM, Golding J, Emond AM (2015) Adverse effects of maternal lead levels on birth outcomes in the ALSPAC study. Br J Obstetr Gynaecol 122: 322–328
- Taylor CM, Golding J, Emond AM (2016) Blood mercury levels and fish consumption in pregnancy: risks and benefits for pregnancy outcomes in a prospective observational birth cohort. Int J Hyg Environ Health 219:513-520
- Golding J, Gregory, S, Iles-Caven Y, Emond A, Hibbeln J, Taylor CM (2017) Maternal prenatal blood mercury is not adversely associated with offspring IQ at 8 years provided the mother eats fish: a British pre-birth cohort study. Int J Hyg Env Health 220:1161-116.
- Taylor CM, Golding J, Emond AM (2016) Moderate prenatal cadmium exposure and adverse birth outcomes: a role for sex-specific differences? Paediatr Perinatal Epidemiol doi: 10.1111/ppe.12318

- Golding J, Gregory, S, Iles-Caven Y, Hibbeln J, Emond A, Taylor CM (2016) Association between prenatal mercury exposure and early child development in the ALSPAC study. NeuroToxicology 53:215-222

Further examples of work relating to developing the understanding of domestic air quality sampling include:

- Brown VM, Crump DR, Gardiner D, Gavin M. Assessment of a passive sampler for the determination of aldehydes and ketones in indoor air. Environmental technology. 1994 Jul 1;15(7):679-85.
